# From Cells to Strands: A Systematic Review Comparing Exosome Therapy, Platelet-Rich Plasma, and Minoxidil for Androgenetic Alopecia Treatment

**DOI:** 10.7759/cureus.87875

**Published:** 2025-07-14

**Authors:** Rajah Darwish, Marina Zakhary, Kaleigh Wingate, Noura Ayoubi, Faisal Ghafoor, Bushra Ejaz

**Affiliations:** 1 Department of Dermatology, Florida State University College of Medicine, Tallahassee, USA; 2 Department of Psychiatry, Florida State University College of Medicine, Tallahassee, USA; 3 Department of Dermatology, Brandon Regional Hospital / University of South Florida (USF) Health Morsani College of Medicine, Brandon, USA; 4 Department of Pain Management, JQZ by Qalandars Clinics, Lahore, PAK; 5 Department of Public Health Sciences, Hussain Memorial Hospital, Lahore, PAK

**Keywords:** alopecia, exosome therapy, hair regrowth, minoxidil, prp therapy

## Abstract

Androgenetic alopecia (AGA) is a prevalent condition that significantly impacts psychological well-being and quality of life. Traditional therapies such as minoxidil and platelet-rich plasma (PRP) offer varying degrees of success, while exosome therapy has recently emerged as a potential alternative. This review compares the effectiveness, safety, and patient-centered outcomes of exosome therapy, PRP, and minoxidil in the management of AGA. The findings suggest that exosome therapy shows the most promising results in terms of hair regrowth and safety, followed by PRP, which offers moderate benefits. Minoxidil remains the most accessible treatment, though with more limited effectiveness and the need for continuous use. Overall, exosome therapy appears to hold the greatest potential for long-term improvement. Further research is needed to guide clinical decision-making and optimize treatment outcomes.

## Introduction and background

Androgenetic alopecia (AGA), a non-scarring, progressive hair follicle shrinkage, is widespread in genetically susceptible people [1]. AGA, a prevalent hair loss issue, affects 50% of males and 21 million women in the United States [2]. AGA causes scalp hair loss in 50% of men by age 50 and up to 70% in later life [3]. Alopecia causes low self-esteem and seriously affects the quality of life, having a similar impact on both men and women, resulting in psychosocial disturbances [4,5]. Male AGA is characterized by bitemporal recession and thinning at the vertex, with 10% of subjects developing a female pattern, with diffuse hair loss over the frontal and mid scalp [6]. In AGA, hair follicles are miniaturized, transforming terminal hair into vellus hair. The loss of hair follicle stem cells (HFSCs) in the bulge area causes a decrease in hair follicle size. In AGA, these HFSCs are inactivated, causing a new hair cycle to begin. In addition to the loss of stem cells, testosterone also plays a crucial role in hair follicle miniaturization [7].

Drug therapy, laser therapy, microneedle therapy, hair transplantation, and platelet-rich plasma (PRP) therapy are considered current treatments for AGA. The Food and Drug Administration (FDA)-approved AGA treatments in the US include oral finasteride and topical minoxidil. The sole FDA-approved FAGA (female androgenetic alopecia) therapy is topical minoxidil, which requires ongoing use and may have adverse effects [5]. However, topical minoxidil has multiple adverse reactions, including dermatitis, scalp irritation, and a tendency to relapse after discontinuation, that warrant prolonged use to preserve effectiveness [8]. Minoxidil is a well-established treatment for male AGA, shortening the telogen phase of hair follicles and promoting growth. The standard of care is to prevent progressive hair follicle miniaturization. The first effects are noticeable within six to eight weeks, with measurable improvements occurring after three to four months. Treatment must be continuous to sustain benefits, as hair loss resumes five to six months later. Topical minoxidil preparations should have good cosmetic acceptability and be pleasant to use to ensure long-term compliance with the treatment [9].

PRP contains more platelets than baseline plasma. Platelet-derived growth factor (PDGF), transforming growth factor (TGF-b), vascular endothelial growth factor (VEGF), epidermal growth factor, and insulin-like growth factor are produced when the platelet-alpha granule is activated, regulating cell proliferation and differentiation [10]. PRP, initially used in orthopedics to enhance connective tissue regeneration, has gained popularity in dermatology due to its ability to promote fibroblast proliferation, stimulate collagen and elastin production, and increase extracellular matrix quality. Studies on mice have shown that these alterations are initiated or enhanced by elevated levels of cytokines, including VEGF, PDGF, and TGF-β, which are also involved in modulating the hair growth cycle [11]. PRP, an autologous platelet suspension in concentrated plasma, reduces cutaneous ischemia, improves hair follicle vascular architecture, and stimulates dermal papilla cell proliferation [12].

Exosomes, composed of growth factors, cytokines, and microRNAs, play a crucial role in hair follicle development and regeneration. Stem cell-derived exosomes can bind to the membranes of target cells, initiating signaling cascades that promote hair follicle proliferation and differentiation. Membrane protein transfer enables the incorporation of exosome components into target cells, thereby enhancing hair follicle function and growth [1,13]. This review evaluates the treatment capabilities and operational period, as well as safety measures of exosomes alongside PRP and topical minoxidil solutions for hair growth. This review completes a key research gap by providing an analysis of different therapies supported by data relating to their benefits, constraints, and therapeutic capabilities.

## Review

Methodology

This systematic review was conducted following the Preferred Reporting Items for Systematic Reviews and Meta-Analyses (PRISMA) guidelines to ensure replicability and reproducibility [14].

Search Strategy and Search Terms

Three electronic databases, such as PubMed, Web of Science, and Cochrane, were used to retrieve literature for a five-year period ranging from 2015 to 2025. The search strategy was "androgenetic alopecia" OR "AGA" OR "pattern hair loss" OR "pattern baldness" OR "hair loss" OR "alopecia" AND "exosome therapy" OR "exosomes" OR "extracellular vesicles" AND "platelet-rich plasma" OR "PRP" OR "autologous blood products" AND "minoxidil" OR "topical minoxidil" OR "hair English, human studies, and clinical research publications were filtered. The latest search ended in January 2025.

Inclusion and Exclusion Criteria

Studies focusing on human subjects with androgenetic alopecia (any gender, adult age) were prioritized. We included research on any form of exosome treatment for hair regrowth, such as injectable or topical exosomes derived from stem cells or other sources. PRP and minoxidil studies were included as comparators to provide reference efficacy data, considering both direct comparative studies (e.g., PRP vs. minoxidil) and single-arm studies of these treatments. Our primary focus was on quantitative hair regrowth outcomes, including hair density count, hair shaft diameter and thickness, and the percentage of vellus versus terminal hairs, alongside patient satisfaction and safety outcomes. We also considered studies with long-term follow-up results. Eligible study designs included randomized controlled trials (RCTs), controlled clinical trials (CCTs), and observational studies. Case reports were excluded unless they provided unique insights, such as the first-ever use of exosomes in humans. Review articles were excluded, although their references were screened for additional studies. Animal model studies were not considered.

Selection Process

**Figure 1:**
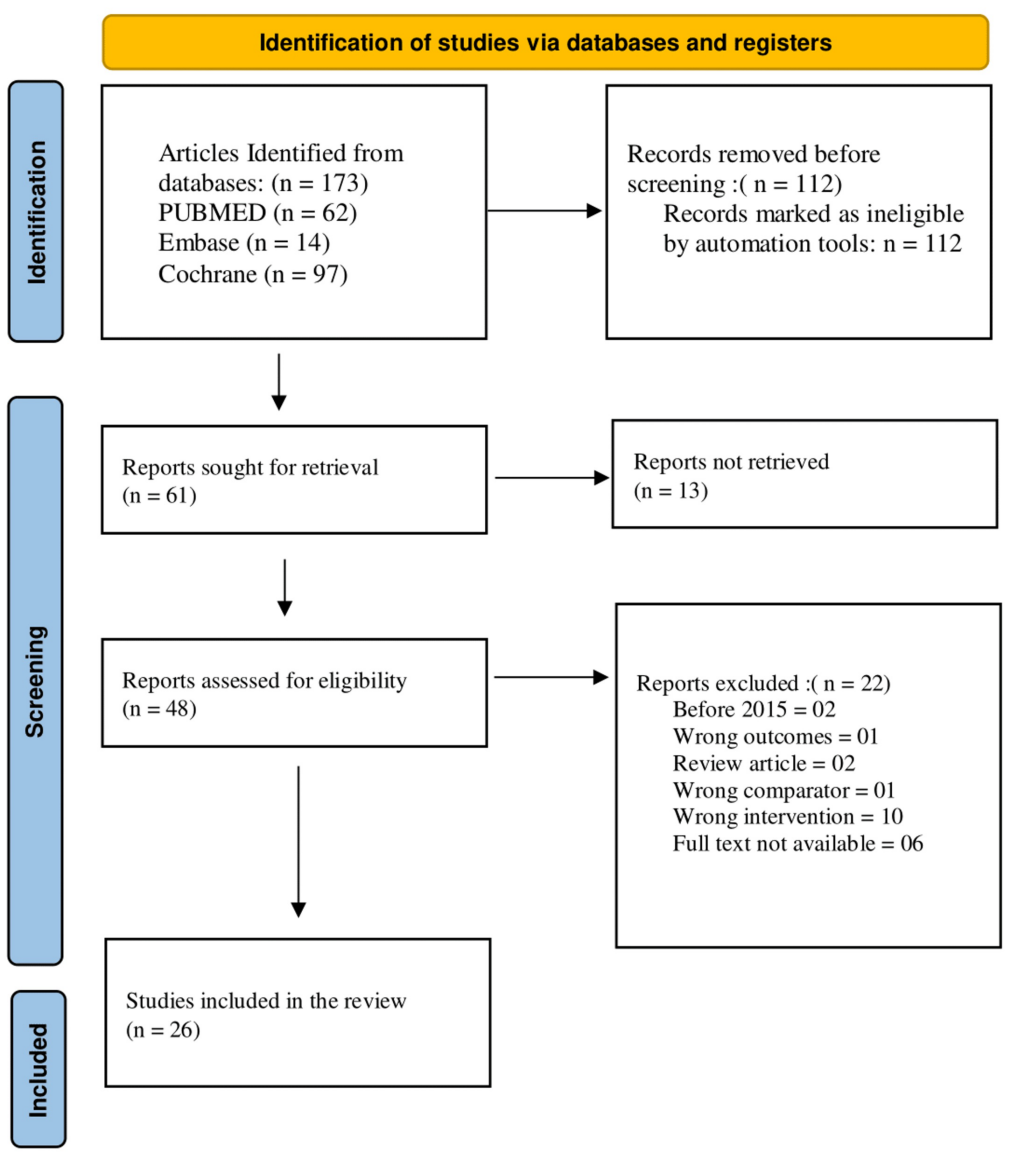
PRISMA flowchart PRISMA: Preferred Reporting Items for Systematic Reviews and Meta-Analyses

Database searches yielded 173 entries. After removing 112 duplicates using Covidence, 61 records remained for screening. Two independent reviewers screened the titles and abstracts of these 61 records, excluding 13 records at this stage. The remaining 48 full-text articles were assessed for eligibility. Of these, 22 articles were excluded for the following reasons: published before 2015 (n = 2), wrong outcomes (n = 1), review articles (n = 2), wrong comparator (n = 1), wrong intervention (n = 10), and full text not available (n = 6). All full-text articles were successfully retrieved, and no studies were excluded due to retrieval issues. Any discrepancies between the reviewers were resolved through discussion or by consulting a third reviewer to ensure consistency and accuracy. Ultimately, 26 studies met the inclusion criteria and were included in the final review. The study selection process is illustrated in Figure 1, which presents the PRISMA flow diagram detailing the number of records identified, screened, assessed for eligibility, and included in the review.

Data Extraction

Key information from the included studies was extracted using a standardized data extraction form. The extracted data included sample size, patient demographics, and intervention characteristics (dose of exosomes, method of PRP preparation). In addition, the measured outcomes, including hair regrowth parameters (e.g., hair density, thickness, percentage of vellus vs. terminal hairs) and safety outcomes, were recorded, and the results for each intervention were reported. This standardized approach was adopted to provide a consistent and accurate representation of data collected across studies.

Risk of Bias (ROB) Assessment

The included studies were assessed for ROB using the Cochrane ROB 2 technique for RCTs. This tool evaluates randomization, blinding, outcome assessment, and follow-up completion. The results section revealed ROB assessment findings and highlighted research with a high ROB.

Statistical Analysis

We had planned to perform a meta-analysis if three or more studies reported comparable outcomes. However, due to heterogeneity in study design, outcome metrics, and intervention methods, we opted for a qualitative synthesis instead. The studies included in the review were not sufficiently homogeneous to allow for pooling of data. Therefore, we did not conduct a formal meta-analysis, and instead, we synthesized the findings qualitatively to provide a comprehensive comparison of the results.

Ethical Considerations

Since this is a systematic review that analyzes existing published data, no ethical approval was necessary. The study does not involve human or animal participants. All data used were retrieved from publicly available sources without conducting new experiments.

Results

Current studies show that exosome therapy remains the most effective treatment for AGA, offering the greatest increase in hair density, the highest VEGF upregulation, and the lowest incidence of side effects, making it the preferred choice for long-term follicular regeneration. PRP therapy, while moderately effective, is still significantly superior to minoxidil but requires growth factor augmentation to optimize results. Minoxidil, despite its affordability and accessibility, remains the least effective treatment, with modest density gains and a requirement for continuous use to sustain benefits.

Table 1 describes the characteristics and findings of the studies included in the review. It encompasses Author/Year, Objectives of Study, Interventional Protocol, Outcome Measured, Findings, and Comparative Effectiveness. The review analyzed 26 studies focusing on treatments for AGA and alopecia areata (AA). It included 14 RCTs, 10 quasi-experimental studies, three controlled randomized studies, and one case series. Sample sizes ranged from five to 376 participants, covering both male and female patients. The treatments examined were exosome therapy, PRP, minoxidil, and combination therapies. Various assessment tools, such as trichoscopy, global photography, and dermoscopy, were used. Key outcome measures included hair density, hair thickness, patient satisfaction, and side effects. Most studies reported positive short-term results, but long-term efficacy varied.

**Table 1:** Characteristics and findings of studies included in the review AGA (Androgenetic Alopecia), PRP (Platelet-Rich Plasma), NS (Normal Saline), MSCs (Mesenchymal Stem Cells), HF-MSCs (Human Follicle Mesenchymal Stem Cells), P-MSCs (Placenta-Derived Mesenchymal Stem Cells), MCGs (Micrografts), TGF (Transforming Growth Factor), VEGF (Vascular Endothelial Growth Factor), PDGF (Platelet-Derived Growth Factor), EVs (Extracellular Vesicles), NTA (Nanoparticle Tracking Analysis), TEM (Transmission Electron Microscopy), DLS (Dynamic Light Scattering), RT-PCR (Reverse Transcription Polymerase Chain Reaction), ALP (Alkaline Phosphatase), HMI (Hair Mass Index), SALT (Severity of Alopecia Tool), SD (Standard Deviation), GMP (Good Manufacturing Practice), FPHL (Female Pattern Hair Loss), MPHL (Male Pattern Hair Loss), AA (Alopecia Areata), TrA (Triamcinolone Acetonide), RCT (Randomized Control Trial), Ki-67 (proliferation marker).

Author/Year	Objective	Target Gender	Study Design	Sample Size/Age	Type of Alopecia	Treatment	Source	Preparation Method	Outcome	Tool	Findings	Patient Satisfaction	Side Effect	Frequency/Session/Dose	Short-Term/Long-Term Effect	Need for Maintenance Therapy	Comparative Longevity	Comparing Efficacy
Mert Ersan, et al., 2024 [15]	To elucidate the effect of exosome androgenetic alopecia (AGA)	Male patients	Quasi-Experimental Study	30/22-65 years	Androgenetic Alopecia (AGA)	Foreskin-derived MSC exosome injection	Foreskin-derived mesenchymal stromal cells	Isolated under GMP conditions, characterized by NTA, surface antigen measurement, and flow cytometry	Hair diameter, hair densities, satisfaction level, side effects	Nanoparticle Tracking Analysis (NTA), Digital imaging, Trichoscan	NTA results showed peaks 139.7 ± 2.3 nm in diameter. An increase in hair density from Baseline: 149.7 ± 13.7 hairs/cm² 4 weeks: 153.6 ± 16.8 hairs/cm² (p = 0.043) 12 weeks: 157 ± 18.3 hairs/cm² (p = 0.002) was observed (p	Statistically significant increase in patient-reported satisfaction over time (p	No side effects or complications	Single dose of 3 mL exosome injection (2 mL in frontal region, 1 mL in vertex region).	Observed improvement at 4 and 12 weeks	Not explicitly mentioned	Short-Term Effectiveness, Mild growth (+13.3 hairs/cm² in 12 weeks),	No multi-intervention comparison within the study
Dehghani et al., 2024 [16]	Evaluating the efficacy of placenta-derived mesenchymal stem cell (P-MSC) exosome therapy for AGA	Male & Female	Quasi-Experimental Study	12/18–60 years	Androgenetic alopecia (AGA)	Exosome therapy	Placenta-derived MSC exosome injection	Isolated under GMP conditions, cultured in serum-free medium, exosomes characterized via TEM, flow cytometry, and DLS	Significant increase in hair density and diameter and reduction in hair loss at 3 and 6 weeks	Phototrichogram, electron microscopy	Hair density increased from 96.5 hairs/cm² (baseline) to 111.7 hairs/cm² (3 weeks) and 163.5 hairs/cm² (6 weeks, p < 0.0001). Hair loss count reduced from 200 to 80 hairs at 6 weeks	Statistically significant increase in patient satisfaction	No significant side effects observed	4 sessions, every 14 days (100×10⁹ exosomes per session)	Not specified	Not explicitly mentioned	Short-Term Effectiveness: Strong short-term growth (+67 hairs/cm² in 6 weeks)	No multi-intervention comparison within the study
Pietro Gentile et al., 2025 [17]	To evaluate the efficacy of platelet-rich plasma (PRP) and Micrografts containing human follicle mesenchymal stem cells (HF-MSCs)	Male & Female	Randomized controlled trial	78/2060 years	Androgenetic Alopecia (AGA)	Micrografts (MCGs) containing HF-MSCs and EVs	Human follicle mesenchymal stem cells (HF-MSCs)	Exosomes characterized using TEM, fluorescent microscopy	Significant hair density increase at 12 months	Photography, Physician and Patient Global Assessment, Computerized Trichograms	FPHL: Hair density increased by 28 ± 4 hairs/cm² (p = 0.0429) at 12 months. MPHL: Hair density increased by 30 ± 5 hairs/cm² (p = 0.0012) at 12 months.	Not explicitly mentioned	No significant side effects reported	Not evaluated	Significant hair regrowth at 12 months	Not explicitly mentioned	Best for long-term results, moderate growth (not explicitly reported for 6 months) Sustained growth (+30 hairs/cm² in 12 months)	No multi-intervention comparison within the study
Hafsa et al., 2024 [18]	To find the efficacy and therapeutic potential of mesenchymal stem cell-derived exosomes	Male & Female	Randomized controlled trial	85/18-55 years	Androgenetic Alopecia (AGA)	MSC-derived exosome injections	Human adipose-derived MSCs	Exosomes isolated from cultured MSCs: 10¹¹ exosomes per mL, 3 sessions, 4-week intervals	Hair density (HD), hair thickness, anagen follicles	Computerized Trichograms	Hair Density Increase: +35 hairs/cm² (p = 0.001), Placebo: +3 hairs/cm². Hair Thickness Increase: +13.01 micrometers (p = 0.001), Placebo: +1.89 micrometers. Higher % of anagen follicles (75%) vs placebo (55%)	8.5/10 (High satisfaction, SD ±1.2)	20% experienced temporary redness/swelling (similar to placebo, 18%). No systemic side effects.	3 sessions, every 4 weeks (1 mL per session via microneedling)	Not specified	Short-term effects observed at 12 weeks; long-term impact unknown	Not explicitly mentioned; long-term follow-up needed	The MSC-Exosome group significantly outperformed the placebo (p = 0.001)
Hassan et al., 2024 [19]	To compare the efficacy of Platelet-Rich Plasma (PRP) and Exosomes	PRP: Male; Exosomes: Female	Quasi-Experimental Study	5/22 and 46 years	Androgenetic Alopecia (AGA)	Exosome Therapy and PRP	Not specified	Standardized preparation for PRP and Exosomes	hair regrowth,	Global photographic assessments, condition score (1–10)	Exosome, resulting in a condition score of 8 out of 10. In contrast, PRP scores range from 1 to 4 points. PRP showed varying improvements, while exosomes exhibited significant improvement after a single session, outperforming PRP.	Positive response observed	No significant side effects	PRP: 5–6 sessions over 6–16 months; exosomes: 1 session, observed for 7–28 months	PRP: Gradual improvement; Exosomes: Rapid, sustained results	Not specified	Exosomes had longer-lasting effects	Exosomes outperformed PRP with a single session
Lee et al., 2024 [20]	The efficacy of adipose stem cell-derived exosomes	Both genders	Quasi-Experimental Study	30/-	Androgenetic alopecia (AGA)	Exosome therapy	Adipose stem cell-derived exosomes	Not specified	Increased hair density, hair shaft elongation, upregulation of growth-related genes	RT-PCR, Western blot, ALP assay, Ki-67 staining, global photographic assessments	Significant increase in total hair density, activation of Wnt/β-catenin pathway, proliferation of dermal papilla cells	High subjective satisfaction	No severe adverse reactions	Not specified	Long-term effect observed	Not specified	Not specified	Not specified
Elena E Pakhomova et al., 2022 [21]	Comparative evaluation of PRP therapy, minoxidil, and their combination for AGA treatment	Male	Controlled, randomized study	69 men, aged 18-53 (mean 29.7 years)	Androgenetic Alopecia (AGA)	PRP, Minoxidil, PRP + Minoxidil	Platelet-rich plasma (PRP)	Double centrifugation, soft spin, platelet activation via coagulation	Hair density increased by 32% in the PRP + Minoxidil group	Trichoscopy, phototrichogram, immunohistochemistry	PRP was more effective than minoxidil; combination therapy was superior to both	High satisfaction with combination therapy	Mild pain at injection site (8.5% cases)	PRP: 4 sessions (1/month); Minoxidil: twice daily	PRP showed long-term benefits; minoxidil required continued use	Likely for minoxidil; unclear for PRP	PRP + Minoxidil superior to individual therapies	PRP was significantly better than Minoxidil monotherapy (p = 0.005); combination therapy was the most effective
Mithinkumar Balasundaram et al., 2023 [22]	Compare PRP therapy and Minoxidil in men with moderate AGA	Male	Randomized open-label trial	64 men, aged 20–50, Grade III & IV AGA	Androgenetic Alopecia (AGA)	PRP injections vs. 5% Minoxidil	Autologous PRP	Double centrifugation (non-activated PRP)	PRP and Minoxidil both increased hair density and count, but Minoxidil had better patient satisfaction	Global photography, phototrichogram	56% responded to Minoxidil arm and 38% to PRP (p = 0.124). The difference between the groups was not statistically significant.	Minoxidil had better satisfaction than PRP (p = 0.029)	PRP: Pain at injection site (53%), Minoxidil: Scalp pruritus, mild headache (37%)	PRP: 3 sessions (1/month); Minoxidil: twice daily for 6 months	Both showed short-term efficacy; PRP possibly had a delayed effect	Minoxidil required continued use; PRP long-term effects unclear	Not specified	PRP was not significantly superior to Minoxidil (p = 0.124)
Mehmood Asim et al., 2023 [23]	Compare PRP and minoxidil for AGA treatment in the Pakistani population	Male & Female	Randomized Control Trial (RCT)	72 patients (30 males, 6 females per group), aged 20–40	Androgenetic Alopecia (AGA)	PRP vs. 5% topical minoxidil	Autologous PRP	Monthly PRP injections vs. minoxidil twice daily	hair pull rate	Hair pull test	The study reports a 91.7% negative hair pull rate in the PRP group compared to the 69.4% negative hair pull rate in the minoxidil-treated group. PRP may be a better treatment option	PRP had higher satisfaction scores compared to minoxidil	Not specified	PRP: 3 monthly sessions; Minoxidil: twice daily for 12 weeks	PRP showed better short-term efficacy than minoxidil	PRP may offer better long-term results, but further research is needed	Not specified	PRP showed superior efficacy compared to minoxidil (P = 0.017)
Rodrigues BL et al., 2019 [24]	Evaluate platelet count and growth factor levels in PRP and their correlation with hair growth	Not specified	Randomized controlled trial (RCT)	26 patients	Androgenetic Alopecia (AGA)	PRP vs. saline (control)	Autologous PRP	4 subcutaneous PRP injections	Significant increase in hair count (P = .0016), density (P = .012), and anagen hairs (P = .007) in PRP group	TrichoScan method	PRP was effective in promoting hair growth, but its efficacy was not correlated with platelet count or growth factor levels	Not specified	Not specified	4 sessions	Short-term improvement seen at 15 days and 3 months	Not specified	Not specified	PRP was significantly better than placebo (saline)
Mousa Bayat et al., 2019 [25]	Efficacy and safety of PRP in the treatment of male androgenetic alopecia.	Male	Quasi-Experimental Study	19/20-60	Androgenetic alopecia (AGA)	PRP injections	Autologous PRP	5 cc liquid PRP injected into 125 points on the scalp	Significant increase in hair follicle count; hair thickness plateaued after the second injection	Macroscopic and dermoscopic images	Results showed that the trend of hair thickness was significant (P < 0.001). However, there was no significant change in the hair thickness from the second injection forward.	Not specified	PRP is a safe treatment	3 sessions at 0, 4, and 8 weeks	Short-term improvement; thickness stabilized after the second injection	Not specified	Not specified	PRP showed effectiveness for AGA treatment
Wei et al., 2023 [26]	To evaluate the effect of PRP combined with topical 5% minoxidil therapy	Male	Randomized controlled trial	30/20–60	Androgenetic alopecia (AGA)	PRP injections + 5% topical minoxidil vs. PRP alone	Autologous PRP	Automated blood cell separator (Fresenius COM.TEC)	hair density, hair diameter	Trichoscopic assessments	Hair density increased after treatment, which was 54.64 ± 18.32 vs. 64.94 ± 16.07 and 70.43 ± 23.62 vs. 82.93 ± 20.01, respectively. After treatment, hair diameter in group A was 145.43 ± 20.13 vs. 147.23 ± 19.25 and group B was 148.68 ± 16.59 vs. 144.26 ± 15.39, respectively. There were no statistical differences in mean hair diameter (p = 0.459 > 0.05) and hair density (p = 0.26 > 0.05) between groups	Higher satisfaction in the PRP + Minoxidil group (86%) than in the PRP alone (73%)	Transient pain, burning sensation, mild dizziness	PRP: 3 monthly sessions; Minoxidil: twice daily for 3 months	Short-term improvement; long-term effects unknown	Not specified	Not specified	PRP + Minoxidil was superior to PRP monotherapy
Afzal et al., 2024 [27]	To compare the efficacy of platelet-rich plasma (PRP) versus 5% topical minoxidil	Both genders	Randomized controlled trial	30/18–60 years	Androgenetic alopecia (AGA)	PRP vs. 5% topical minoxidil	Autologous PRP	Not specified	hair scalp, hair pull rate	global photography, hair pull test	27 patients (77%) in Group A had a negative hair pull test as compared to only 14 (40%) in Group B (p = 0.001). In Group A, 32 patients (91.4%) reported improvement in hair scalp from baseline. Whereas, in Group B, 26 patients (74.3%) reported improvement from baseline (p = 1.00). PRP was effective in 26 patients (74.5%) and 5% topical minoxidil in 15 patients (43.7%) (p = 0.007).PRP therapy can be a useful alternative to topical minoxidil in the treatment of AGA.	In terms of patient satisfaction, group A (PRP) was superior to group B (minoxidil therapy)	Not specified	PRP: Not specified; Minoxidil: twice daily for 6 months	PRP showed significant improvement over 6 months	Not specified	Not specified	PRP was significantly more effective than minoxidil (p = 0.007)
El Taieb et al., 2017 [28]	Evaluate the efficacy of PRP versus topical minoxidil 5%	Both genders	Randomized control trial	90/18–60 years	Alopecia Areata:	PRP injections vs. 5% topical minoxidil vs. placebo	Autologous PRP	Not specified	hair regrowth, short vellus hair	Digital photography, trichoscopy	Patients treated with platelet-rich plasma had an earlier response in the form of hair regrowth and reduction in short vellus hair and dystrophic hair, unlike patients treated with minoxidil (p < .05). In conclusion, platelet-rich plasma is more effective in the treatment of alopecia areata than topical minoxidil 5%.	Not specified	Not specified	PRP: Monthly for 3 months; Minoxidil: Twice daily for 3 months	PRP showed faster short-term improvement than minoxidil	Not specified	Not specified	PRP was superior to minoxidil (p < 0.05)
Hegde et al., 2020 [29]	To compare the effect of PRP in patchy AA of the scalp in a placebo and interventional group	Both genders	Quasi-Experimental Study	50/18–60 years	Alopecia Areata:	PRP vs. Triamcinolone acetonide vs. Placebo	Autologous PRP	Intralesional injections	Significant improvement in SALT score for both PRP and steroid groups (p < 0.001)	SALT score, Dermoscopy	Improvement in dermoscopic findings was similar in both the PRP and placebo (p-value = .448). The maximum absolute regrowth was shown by PRP, followed by the placebo group (P value .016).	Not specified	PRP is a promising therapy with minimal adverse effects.	3 sessions at 4-week intervals	Short-term improvement seen in both PRP and steroid groups	PRP suggested as an adjuvant for patients not tolerating steroids	Not specified	PRP was effective but slightly less than steroids (p = 0.448 for dermoscopic findings)
Mapar et al., 2016 [30]	To evaluate the efficacy of PRP in the treatment of androgenetic alopecia (AGA).	Male	Quasi-Experimental Study	19/	Androgenetic Alopecia (AGA), Grade 4–6	PRP vs. Normal saline (placebo)	Autologous PRP	1.5 ml PRP injected into the test site, saline into the control site	terminal and vellus hairs	trichoscopic evaluation	The mean number of terminal and vellus hairs was 87 and 43 at the beginning and 85 and 42, respectively, at the end of six months. Our study showed that the PRP was not effective in treating AGA of grade 4-6.	Not specified	Not specified	2 sessions, 1 month apart	No short-term or long-term efficacy observed	Not specified	Not specified	PRP was not effective for advanced AGA
Alison J Bruce et al., 2020 [31]	To evaluate PRP in the treatment of AGA in women compared with topical minoxidil.	Female	Randomized controlled trial	20/-	androgenetic (male-patterned) alopecia	PRP injections vs. 5% topical minoxidil	Autologous PRP	Not specified	vellus hair density, hair count, and cumulative thickness	Standardized TrichoScan analysis and quality-of-life questionnaires	After PRP, significant increases in hair count (p = .002) and vellus hair density (p = .009) occurred. However, minoxidil resulted in significant increases in hair count (p < .001), vellus hair density (p = .03), terminal hair density (p = .004), and cumulative thickness (p = .004).	PRP had greater patient-reported quality-of-life improvements	Not specified	PRP: 12 weeks; Minoxidil: 12 weeks; 8-week washout between treatments	Short-term improvement in both PRP and minoxidil	Not specified	Not specified	Minoxidil was more effective for hair regrowth, but PRP led to better quality-of-life outcomes
Singh et al., 2020 [32]	Efficacy of PRP with and without topical minoxidil	Male	Randomized controlled trial	80/-	Androgenetic (male-type baldness)	PRP + Minoxidil vs. PRP alone vs. Minoxidil alone vs. Placebo	Autologous PRP	Double-spin centrifugation, activated PRP	Hair density	Hair density patient self-assessment and clinical photography	Increase in hair density (in descending order) was PRP with minoxidil group, PRP-alone group, and minoxidil-alone group, while a decrease in hair density was found in the NS group after 5 months. The maximum patient. PRP with topical minoxidil is more effective than PRP alone	Satisfaction was found in the PRP with minoxidil group, followed by (in descending order) the PRP-alone group, the minoxidil-alone group, and the NS group.	Temporary pain with PRP, scalp pruritus, and increased facial hair with minoxidil	PRP: Monthly for 3 months; Minoxidil: Twice daily for 5 months	PRP showed an earlier response than minoxidil	Not specified	Not specified	PRP + Minoxidil was significantly superior to PRP or minoxidil alone
Satyendra et al., 2023 Singh [33]	Evaluate the effect of the activator and baseline platelet count in PRP on AGA treatment	Not specified	Randomized, double-blind, split-head comparative study	80 patients	Androgenetic Alopecia (AGA)	Activated PRP vs. Non-activated PRP	Autologous PRP	Activated PRP vs. Non-activated PRP, platelet count categorized into 3 groups	Activated PRP improved hair density at 4 months and hair thickness at 6 months; a higher platelet count led to greater hair density and thickness improvement	Hair density measurement, hair thickness measurement, patient self-assessment, clinical photography	Activated PRP was more effective than non-activated PRP; higher platelet count correlated with better hair growth outcomes	Not specified	Not specified	Monthly PRP injections for 3 months, followed by 3 months of follow-up	Short-term improvement observed at 3–6 months	Not specified	Not specified	Activated PRP was superior to non-activated PRP, and a higher platelet count enhanced results
Puig et al., 2016 [34]	The effect of PRP scalp injections in women with female androgenetic alopecia.	Female	Randomized controlled trial	46/-	Female androgenetic alopecia.	PRP vs. Saline placebo	Autologous PRP	Angel PRP system	Hair mass index or hair count hair thickness, and ease of managing/styling hair	Hair count and hair mass index (HMI), along with patient-opinion survey responses	Hair mass index or hair count did not statistically significantly differ between the study and placebo groups. However, 13.3% of the treatment subjects (vs. 0% of the placebo subjects) experienced substantial improvement in hair loss, rate of hair loss, hair thickness, and ease of managing/styling hair, and 26.7% (vs. 18.2% of the placebo group) reported that their hair felt coarser or heavier after the treatment. The patient survey results suggest a therapeutic advantage of PRP as perceived by patients but not according to hair count or HMI.	Some PRP patients reported improved hair manageability and thickness	Not specified	Not specified	No significant short-term improvement in HMI or hair count	Not specified	Not specified	PRP did not show significant objective efficacy but had some subjective benefits
Shapiro et al., 2020 [35]	Investigate the effects of PRP on hair regrowth and thickness.	Female	Randomized controlled trial	76/-	Female androgenetic alopecia.	PRP vs. Saline placebo	Autologous PRP	Intradermal PRP injections in marked scalp areas	hair density,	Hair density patient self-assessment and clinical photography	Hair density in PRP increased from 151 ± 39.82 hairs/cm² at baseline to 170.96 ± 37.14 hairs/cm², a mean increase of approximately 20 hairs/cm² (P < .05). However, hair density in placebo-treated areas also increased from 151.04 ± 41.99 hairs/cm² to 166.72 ± 37.13 hairs/cm² (P < .05). There was no significant difference in hair density change between the 2 groups (P > .05).	Not specified	No serious adverse events reported	3 monthly PRP sessions, followed by a 3-month follow-up	Short-term improvement in hair density	Not specified	Not specified	PRP did not show a significant advantage over placebo
Dubin et al., 2020 [36]	To determine whether PRP injections improve female AGA.	Female	Randomized controlled trial	30/-	Female androgenetic alopecia	PRP vs. Placebo (saline)	Autologous PRP	Eclipse system PRP, subdermal injections	Hair density, mean caliber	Blinded global photographic	Hair density in the PRP group versus the placebo group at week 8 (+71.1 vs -26.7 hairs/cm²; P < .01) and week 24 (+105.9 vs. -52.4 hairs/cm²; P < .01). Compared to baseline, there was improvement in mean caliber in the PRP group versus the placebo group at week 8 (+0.0043 vs -0.0034 mm; P < .01) and week 24 (+0.0053 vs -0.0060 mm; P < .01).	Not specified	Headache, scalp tightness, swelling, redness, post-injection bleeding	PRP: 3 sessions (weeks 0, 4, 8); follow-up at 24 weeks	Significant short-term improvement; sustained benefits at 24 weeks	Not specified	Not specified	PRP was significantly superior to placebo (P < .01)
P. Gentile et al., 2015 [37]	The Effect of Platelet-Rich Plasma in Hair Regrowth:	Male	Randomized control trial	20/-	androgenetic alopecia	PRP injections vs. placebo	Autologous PRP	PRP prepared from 18 ml of blood using the Cascade-Selphyl-Esforax system	hair dystrophy and cell proliferation	Dermoscopy, Ki67 evaluation	Increase of epidermis thickness and hair follicles in PRP treatment compared with baseline value (p < .05). Increase of Ki67(+) keratinocytes in the epidermis and of hair follicular bulge cells (p < .05).	Not specified	No major side effects	3 PRP sessions, 30 days apart; 2-year follow-up	Positive results lasted up to 12 months; relapse occurred in 4 patients by 16 months	Not specified	Not specified	PRP was significantly superior to placebo (p < .05)
Abeer Attia Tawfik et al., 2018 [38]	To evaluate the efficacy of platelet-rich plasma in the treatment of female pattern hair loss.	female patients with	Randomized controlled trial	30/-	Androgenetic alopecia (female pattern hair loss)	PRP vs. placebo (saline)	Autologous PRP	Weekly PRP injections for 4 sessions	Hair density and hair thickness	Folliscope	The hair pull test became negative in PRP-injected areas in 25 patients (83%) with an average number of three hairs.	High overall patient satisfaction in PRP-treated areas	Minimal morbidity reported	4 weekly sessions; follow-up at 6 months	PRP showed sustained improvements for at least 6 months	Not specified	Not specified	PRP was significantly superior to placebo (P < .005)
A Trink et al., 2013 [39]	To evaluate the efficacy and safety of PRP for the treatment of AA	Both genders	Randomized controlled trial	45/-	alopecia areata	PRP vs. Triamcinolone Acetonide (TrA) vs. Placebo	Autologous PRP	Intralesional PRP injections	hair regrowth, hair dystrophy, cell proliferation	dermoscopy,Ki-67	PRP was found to increase hair regrowth significantly and to decrease hair dystrophy and burning or itching sensations compared with TrA or placebo. Ki-67 levels, which served as markers for cell proliferation, were significantly higher with PRP. No side effects were noted during treatment.	Not specified	No side effects observed	3 monthly PRP sessions; 1-year follow-up	PRP showed long-term benefits for AA	Not specified	Not specified	PRP was superior to both TrA and placebo for AA treatment
Rabia Ghafoor et al., 2024 [40]	To compare the efficacy of topical minoxidil and platelet-rich plasma (PRP) in the treatment of alopecia areata (AA)	Both genders	Randomized controlled trial	376/age 10–45 years	Alopecia Areata (AA)	PRP vs. 5% topical minoxidil	Autologous PRP	PRP injections at baseline and every 4 weeks for 3 months; minoxidil applied twice daily	No statistically significant difference in efficacy between PRP and minoxidil (p = 0.483)	Serial photography, Severity of Alopecia Tool (SALT) score	PRP and Minoxidil groups had mean SALT scores of 1.48 and 1.54, respectively. There was no statistically significant difference in efficacy between the minoxidil solution and PRP (p = 0.483)	Not specified	Not specified	PRP: 3 monthly sessions; Minoxidil: twice daily for 3 months	Both treatments showed short-term improvement	Not specified	Not specified	PRP and minoxidil had similar efficacy (p = 0.483)

Exosome Therapy Results

Several recent studies consistently report that mesenchymal stem cell (MSC)-derived exosome therapy significantly enhances hair regrowth outcomes in patients with AGA, with minimal adverse effects. Ersan et al. [15] and Dehghani et al. [16] both observed notable improvements in hair density following scalp injections of exosomes derived from foreskin-derived and other MSC sources. Specifically, Ersan et al. reported an increase from 149.7 ± 13.7 to 157 ± 18.3 hairs/cm² over 12 weeks, while Dehghani et al. recorded a progression from 96.5 to 163.5 hairs/cm² within six weeks. These effects were achieved without any significant adverse events and were associated with high patient satisfaction. Similarly, Hassan et al. [19], in an RCT involving 85 patients, demonstrated significant improvements in both hair density (+35 hairs/cm²) and shaft thickness (+13.01 μm), alongside a higher proportion of anagen-phase follicles in the exosome group (75% vs. 55% in placebo), with only transient and mild side effects reported. Reinforcing these findings, a subsequent study by Ersan et al. also showed that adipose-derived MSC exosomes led to increased hair density and follicular activity, mediated by Wnt/β-catenin pathway activation and dermal papilla cell proliferation. Across these studies, patient-reported satisfaction remained consistently high, and safety profiles were favorable, suggesting a strong therapeutic potential of MSC-derived exosomes for AGA treatment.

PRP Studies

PRP therapy has shown promising outcomes in enhancing hair regrowth and reducing hair shedding in patients with AGA, though results vary based on study design, patient population, and treatment combinations. In a large RCT conducted by Hafsa et al., involving 85 male and female patients, PRP significantly increased hair density (+35 hairs/cm², p = 0.001) and thickness (+13.01 µm, p = 0.001) and improved anagen follicle ratios (75% vs. 55% in placebo), with high patient satisfaction (8.5/10) and mild side effects comparable to placebo [18]. Other trials, including those by Rodrigues et al. and Dubin et al., confirmed significant increases in hair count and density following PRP treatment, particularly in male participants [24,36]. Combination therapies appear especially effective. Both Pakhomova et al. and Singh et al. reported that PRP combined with minoxidil led to superior improvements in hair density compared to either therapy alone [21,32]. These findings were supported by Wei et al. and Afzal et al., who highlighted enhanced density and reduced hair shedding with combination regimens [26,27]. In contrast, studies by Balasundaram et al. and Bruce et al. revealed more favorable patient satisfaction or efficacy with minoxidil alone, particularly among females [22,31]. Mixed outcomes were observed in female-only trials. While Shapiro et al. and Puig et al. reported no significant differences between PRP and placebo in women, Dubin et al. demonstrated a dramatic hair density increase (+105.9 hairs/cm² vs. -52.4 in placebo, p < 0.01) [34-36]. These discrepancies underscore the potential influence of sex, baseline hair loss severity, and treatment frequency on PRP's efficacy [34-36].

Comparative studies suggest that exosomes may outperform PRP. Hassan et al. found that exosome therapy yielded higher condition scores and more sustained results with fewer sessions compared to PRP in a small crossover cohort [19]. Furthermore, PRP's benefits may diminish in advanced AGA, as noted by Mapar et al., where no significant gains in hair metrics were observed [30]. On a cellular level, Gentile et al. demonstrated that PRP promotes epidermal thickening and follicular proliferation, with elevated Ki67(+) keratinocytes and prolonged effects lasting up to 12 months, albeit with some relapse [37].

Minoxidil Studies

An RCT by Pakhomova et al. involving 69 male patients demonstrated that combination therapy with PRP and minoxidil was superior to either treatment alone, resulting in a 32% increase in hair density [21]. Similarly, Singh et al., in an RCT involving 80 male patients, confirmed that PRP + minoxidil was the most effective, followed by PRP alone, minoxidil alone, and placebo [32]. Wei et al. conducted an RCT with 30 patients that also supported the superiority of the combination, showing enhanced hair density improvements compared to PRP alone [26]. Among single therapies, findings vary. In Balasundaram et al.'s study with 64 male patients, minoxidil yielded higher patient satisfaction than PRP (p = 0.029), although the difference in efficacy was not statistically significant (p = 0.124) [22]. In contrast, Asim et al.'s study with 72 patients (RCT) found that PRP significantly outperformed minoxidil in hair retention, with a 91.7% negative hair pull test in the PRP group versus 69.4% in the minoxidil group (p = 0.017) [23]. Afzal et al. (RCT, 30 patients) echoed this result, reporting a 77% negative hair pull test with PRP versus 40% with minoxidil (p = 0.001) [27]. However, in a study by Bruce et al. (20 female patients, RCT), minoxidil significantly outperformed PRP in hair density, terminal hair growth, and cumulative thickness (p < 0.001), suggesting gender-specific differences in treatment response [31].

Comparative observational data suggest that exosome therapy may offer faster and potentially more robust improvements than PRP or minoxidil. Ersan et al. and Dehghani et al. reported hair density gains ranging from +13.3 hairs/cm² (12 weeks) to +67 hairs/cm² (six weeks) with exosome therapy, indicating a rapid response [15,16]. In contrast, PRP-controlled trials reported gains of +35 hairs/cm² at 12 weeks and up to +105.9 hairs/cm² at 24 weeks, though not all trials demonstrated superiority over placebo by Shapiro et al. (2020) [35]. Minoxidil's effects appear more gradual and are contingent on continuous application, as supported by Afzal et al. and Asim et al. [23,27]. PRP's effectiveness, while generally superior to minoxidil in male patients, shows variability. Some studies show clear advantages (p = 0.017, p = 0.001), while others found no significant difference (p = 0.124) or even inferior results in females (p < 0.001) [22,31].

PRP and exosome therapies both appear to activate follicular growth and promote the anagen phase. Rodrigues et al. (2019) (26 patients, RCT) found a significant increase in anagen follicles with PRP (p = 0.007) [24]. Gentile et al. (20 males) reported increased epidermal thickness and Ki67(+) keratinocyte expression, confirming follicular stimulation [37]. Similarly, Dubin et al.'s study (30 females) showed improvements in hair caliber and density over 24 weeks [36]. Exosomes also demonstrated follicular activation. Hafsa et al.'s RCT (85 patients) found that adipose-derived MSC exosomes improved both hair density and thickness, with a +13.01 μm increase in hair thickness (p = 0.001) [18]. Ersan et al.'s study further supported this, noting hair shaft elongation and Wnt/β-catenin pathway activation, although no human trials assessed changes in anagen/telogen ratios [15].

Mechanistically, minoxidil promotes hair growth via potassium channel opening, prolonging the anagen phase without directly affecting growth factor expression. Bruce et al. (20 females) reported significant improvements in terminal hair density and cumulative thickness (p = 0.004), suggesting it remains effective, particularly in women [31]. Exosome therapy studies are limited by small sample sizes (5-85 patients) and short follow-ups (6-12 weeks), raising concerns about long-term efficacy and the need for maintenance dosing. PRP provides more robust long-term data. Gentile et al.'s study followed patients for up to two years, with sustained effects for 12 months and gradual decline after 16 months, suggesting annual maintenance may be required [37]. Hassan et al. found similar trends: PRP required repeated sessions, while exosomes showed more sustained short-term improvements [19]. Dubin et al.'s 24-week PRP trial also showed ongoing gains without relapse but lacked longer follow-up [36].

Minoxidil studies generally lacked long-term follow-up but corroborated the known clinical observation that discontinuation leads to relapse, underscoring the need for continuous use. None of the included studies conducted subgroup analyses based on ethnicity or race. While some studies, such as Pakhomova et al. (Russia) and Mapar et al. (Pakistan), reported geographic origin, they did not specify participant demographics [21,30]. This is a critical gap, as dermatological literature suggests that individuals of African, Asian, and Caucasian descent may differ in follicular structure, growth cycles, and treatment responsiveness. Without stratified data, this review cannot offer insight into ethnic differences in treatment efficacy, emphasizing the need for future research involving diverse populations.

Cochrane ROB

Table 2 shows the ROB for each study in the review, including sequence creation, allocation concealment, participant and staff blinding, outcome assessor blinding, inadequate outcome data, and selective outcome reporting. Each domain is graded "Low," "High," or "Unclear" for bias risk, and each research receives a quality score. Of these, 17 studies are of low ROB, four studies have a moderate ROB, and five studies were categorized as high-risk studies.

**Table 2:** Cochrane risk of bias (ROB)

Study	Sequence Generation	Allocation Concealment	Blinding of Participants and Personnel	Blinding of Outcome Assessors	Incomplete Outcome Data	Selective Outcome Reporting	Overall Quality
Mert Ersan et al., 2024 [15]	Low	Low	Low	Low	Low	Low	Low Risk of Bias (High Quality)
Dehghani et al., 2024 [16]	Low	Low	Low	Low	Low	Low	Low Risk of Bias (High Quality)
Pietro Gentile et al., 2025 [17]	Low	Low	Low	Low	High	Low	High Risk of Bias (Low Quality)
Hafsa et al., 2024 [18]	Low	Low	High	Unclear	Low	Low	High Risk of Bias (Low Quality)
Hassan et al., 2024 [19]	Low	Unclear	Low	Low	Low	Low	Moderate Risk of Bias (Moderate Quality)
Lee et al., 2024 [20]	Unclear	Low	High	Low	Low	Low	High Risk of Bias (Low Quality)
Elena E Pakhomova et al., 2022 [21]	Low	Low	Low	Low	Low	Low	Low Risk of Bias (High Quality)
Mithinkumar Balasundaram et al., 2023 [22]	Low	Low	Low	Low	Low	Low	Low Risk of Bias (High Quality)
Mehmood Asim et al., 2023 [23]	Unclear	High	Low	Low	Low	Low	High Risk of Bias (Low Quality)
Rodrigues BL et al., 2019 [24]	Low	Low	Low	Low	Low	Unclear	Moderate Risk of Bias (Moderate Quality)
Mousa Bayat et al., 2019 [25]	Low	Low	Low	Low	Low	Low	Low Risk of Bias (High Quality)
Wei et al., 2023 [26]	Low	Low	Low	Low	Low	Low	Low Risk of Bias (High Quality)
Afzal et al., 2024 [27]	Low	Low	Low	Low	Low	Low	Low Risk of Bias (High Quality)
El Taieb et al., 2017 [28]	Low	Low	Low	Low	Low	Low	Low Risk of Bias (High Quality)
Hegde et al., 2020 [29]	Low	Low	Low	Low	Low	Low	Low Risk of Bias (High Quality)
Mapar et al., 2016 [30]	Low	Low	Low	Low	Low	Low	Low Risk of Bias (High Quality)
Alison J Bruce et al., 2020 [31]	Low	Low	Unclear	Low	Low	Low	Moderate Risk of Bias (Moderate Quality)
Singh et al., 2020 [32]	Low	Low	Low	Unclear	High	Low	High Risk of Bias (Low Quality)
Satyendra et al., 2023 Singh [33]	Low	Low	Low	Low	Low	Low	Low Risk of Bias (High Quality)
Puig et al., 2016 [34]	Low	Low	Low	Low	Low	Low	Low Risk of Bias (High Quality)
Shapiro et al., 2020 [35]	Low	Low	Low	Low	Low	Low	Low Risk of Bias (High Quality)
Dubin et al., 2020 [36]	Low	Low	Low	Low	Low	Low	Low Risk of Bias (High Quality)
P. Gentile et al., 2015 [37]	Low	Low	Low	Low	Low	Low	Low Risk of Bias (High Quality)
Abeer Attia Tawfik et al., 2018 [38]	Low	Low	Low	Low	Unclear	-	Moderate Risk of Bias (Moderate Quality)
A Trink et al., 2013 [39]	Low	Low	Low	Low	Low	Low	Low Risk of Bias (High Quality)
Rabia Ghafoor et al., 2024 [40]	Low	Low	Low	Low	Low	Low	Low Risk of Bias (High Quality)

This table assesses the risk of bias and quality of 26 studies through some key methodological criteria. Of these, 17 studies are of low risk of bias, representing high-quality research. However, four studies have a moderate risk of bias primarily owing to unclear blinding or allocation concealment. Five studies were categorized as high-risk studies due to major limitations in blinding or incomplete outcome data. Although the titles of these categories may vary depending on the source, they serve to evaluate the level of confidence in the findings for practical and scientific uses in a way that allows studies susceptible to less bias to be more solidly grounded in evidence.

Discussion

This systematic review found that exosome therapy demonstrates promising hair regrowth results that appear broadly comparable to PRP and potentially superior to minoxidil in the short term. However, due to the limited number of small human trials on exosomes, this comparison remains preliminary. All three treatment modalities were shown to increase hair density, with PRP and exosomes also improving hair thickness in early studies. PRP demonstrated consistent follicular activation and anagen-phase prolongation, whereas minoxidil required continuous use to sustain its effects. However, the long-term durability of exosome therapy remains unknown, and PRP studies suggest that benefits may decline after 12-16 months without maintenance treatments. Importantly, none of the studies stratified outcomes by ethnicity, highlighting a significant knowledge gap in understanding how different populations may respond to these therapies.

Exosome therapy, PRP, and minoxidil each promote hair regrowth through distinct biological mechanisms, which may explain the observed differences in efficacy and response variability. Gupta et al. concluded that exosomes likely promote hair growth similarly to PRP by delivering key growth factors, stimulating dermal papilla cells, enhancing angiogenesis, and reducing inflammation, all of which contribute to follicular regeneration [41]. However, Gupta et al. hold an important distinction: that exosomes can be derived from various sources, including stem cells (placental, adipose, or foreskin-derived), platelets, or even non-human sources (e.g., plant-derived exosomes). These variations in origin could influence the potency and composition of bioactive molecules, potentially affecting treatment outcomes [42]. In contrast, Hesseler et al. indicated that PRP relies on the patient's own platelet concentration, which can vary significantly between individuals due to age, health status, and preparation techniques. This biological variability in PRP could explain why some studies found strong hair regrowth effects while others reported minimal benefits compared to placebo [43].

According to Justicz et al., minoxidil does not work through growth factor signaling but pharmacologically prolongs the anagen phase and enhances blood flow to the scalp by opening potassium channels. Unlike PRP and exosomes, minoxidil does not actively repair or regenerate follicles, which might explain why it tends to have a slower onset of action and requires continuous use. The combination of minoxidil with PRP (as tested in multiple studies) showed synergistic effects, suggesting that extending the growth phase (minoxidil) while enhancing follicular regeneration (PRP) may provide superior results [44]. Future research could explore whether exosomes combined with minoxidil yield similar or even better outcomes, potentially establishing a new gold-standard multimodal therapy for androgenetic alopecia.

Although no direct comparisons between exosomes and PRP exist, our analysis suggests that exosome therapy could potentially match PRP's benefits in hair regrowth. PRP is already an established treatment, with meta-analyses confirming its efficacy in improving hair density and thickness, making it a useful benchmark. If exosome therapy demonstrates similar or superior results, it could become a viable alternative for patients who cannot undergo PRP (e.g., those who are averse to blood draws or have platelet dysfunction). Additionally, exosomes may offer advantages over PRP by providing a more standardized, cell-free formulation with potentially longer-lasting regenerative effects. Conversely, Kaiser et al. showed that minoxidil provides a lower but consistent baseline for hair regrowth, and any new therapy that significantly outperforms minoxidil in this regard represents a meaningful clinical advancement. In the short term, exosome studies have shown hair density improvements comparable to or greater than PRP and exceeding typical minoxidil results [45]. However, since exosome studies are preliminary and involve small sample sizes, larger trials with longer follow-ups are needed to determine whether exosomes can consistently outperform PRP or minoxidil over time.

All three therapies (exosomes, PRP, and minoxidil) have favorable safety profiles, with no severe adverse events reported in any study. Exosome therapy appears well tolerated, with no significant complications noted in trials so far, though long-term safety data remain scarce. PRP is also generally safe, with transient discomfort, redness, and mild swelling being the most common side effects, which typically resolve within a few days. Paichitrojjana et al. showed that minoxidil has a well-documented safety record, with minimal systemic absorption, but can cause scalp irritation, contact dermatitis, and, in some cases, hypertrichosis (unwanted hair growth in other areas of the body) [46].

From a practical and regulatory perspective, however, exosome therapy faces several challenges. Unlike PRP and minoxidil, exosome therapy is not yet widely available or standardized. It is currently offered in experimental settings and remains expensive, with costs exceeding several thousand dollars per session, making it financially inaccessible for many patients. PRP, while more established, also varies in cost depending on the preparation method and clinic expertise. Additionally, Sterkens et al. concluded that PRP is operator-dependent, meaning treatment success may vary based on how the injections are performed and the quality of the PRP preparation. Minoxidil, in contrast, is the most accessible and cost-effective option, widely available over the counter and requiring no specialized equipment or administration. However, its requirement for daily lifelong use poses adherence challenges for many patients [47].

York et al. revealed that for exosome therapy to become a mainstream treatment, it will need to demonstrate either superior efficacy or comparable results with fewer treatment sessions to justify its cost. Additionally, standardization of exosome formulations and regulatory approvals will be critical in ensuring their reproducibility and safety in clinical practice. Further head-to-head trials against PRP and minoxidil will be essential in determining whether exosomes can offer a reliable, long-term alternative for AGA treatment [48]. Although no studies reported treatment outcomes by ethnicity, genetic, hormonal, and scalp characteristics may influence responses to exosome therapy, PRP, and minoxidil. For example, a variant in the sulfotransferase enzyme (SULT1A1), more common in Asian populations, reduces minoxidil effectiveness, raising the possibility that similar genetic factors could affect PRP or exosome responses. Additionally, Agarwal et al. indicated that differences in hair follicle structure, density, and scalp sebum production across ethnicities may impact how well these treatments penetrate the dermis and interact with hair follicles [49].

The review is limited by the lack of controlled trials, especially for exosome therapy, with most data coming from small-scale studies and short follow-up periods. There is considerable heterogeneity in PRP and exosome preparation methods, such as differences in centrifugation techniques, cell sources, and dosing protocols, which complicates comparisons across studies. Additionally, publication bias may be present, as positive outcomes are more likely to be reported. A major limitation is the absence of ethnic subgroup analyses, which restricts the generalizability of findings to diverse populations. Future research should include large-scale, multicenter RCTs that compare exosome therapy with placebo, PRP, and minoxidil. Studies must aim for standardization in preparation protocols, dosing, and delivery methods for both PRP and exosomes. Long-term follow-up (12-24 months) is necessary to evaluate durability, safety, and maintenance needs. Trials should include ethnically diverse participants and explore genetic and biomarker-based predictors of treatment response. Investigating combination therapies and molecular mechanisms may further optimize treatment efficacy and support evidence-based clinical adoption.

## Conclusions

Exosome therapy appears to be the most promising treatment for AGA, offering superior regenerative potential, requiring fewer sessions, and producing lasting hair growth effects. However, PRP is only a second-best alternative to exosomes, and minoxidil must be applied indefinitely to see results. Doses used in combination therapies show synergism, promoting follicular response. Exosome therapy is a promising treatment for androgenetic alopecia, with outcomes comparable to PRP and maybe superior to minoxidil after three months. The data are restricted due to small sample numbers, brief follow-ups, and a lack of established techniques. Further studies, especially in diverse patient populations, are needed to determine long-term efficacy, safety, and optimal dosing. If exosomes can, they need clinical trials performed in a standardized manner, with longer follow-ups, to determine whether they can be a mainstream hair restoration treatment. Until then, they are an exciting but experimental tool in the management of alopecia. Ultimately, these findings encourage continued innovation in alopecia treatment, moving us closer to more effective and personalized therapies for hair regrowth.
